# Comparative Analysis of Canonical Inflammasome Activation by Flow Cytometry, Imaging Flow Cytometry and High-Content Imaging

**DOI:** 10.1007/s10753-024-02141-z

**Published:** 2024-09-10

**Authors:** Nico Wittmann, Sander Bekeschus, Doreen Biedenweg, Daniela Kuthning, Christopher Pohl, Jana Gramenz, Oliver Otto, Lukas Bossaller, Almut Meyer-Bahlburg

**Affiliations:** 1 Pediatric Rheumatology, Department Pediatric and Adolescent Medicine, University Medicine, University of Greifswald, 17475 Greifswald, Germany; 2 ZIK Plasmatis, Leibniz Institute for Plasma Science and Technology (INP Greifswald), Felix-Hausdorff-Straße 2, 17489 Greifswald, Germany; 3 Clinic and Policlinic for Dermatology and Venerology, Rostock University Medical Center, Strempelstraße 13, 18057 Rostock, Germany; 4 Institute for Physics, University of Greifswald, Greifswald, Germany; 5 Department of General Surgery, Visceral, Thoracic and Vascular Surgery, University Medical Center Greifswald, Greifswald, Germany; 6 Section of Rheumatology, Department of Medicine A, University Medicine, University of Greifswald, Greifswald, Germany

**Keywords:** ASC (apoptosis-associated speck-like protein containing a caspase-recruitment domain), Speck, Monocytes, Peripheral blood mononuclear cell, Flow cytometry

## Abstract

Inflammasome activation occurs in various diseases, including rare diseases that require multicenter studies for investigation. Flow cytometric analysis of ASC speck^+^ cells in patient samples can be used to detect cell type-specific inflammasome activation. However, this requires standardized sample processing and the ability to compare data from different flow cytometers. To address this issue, we analyzed stimulated and unstimulated PBMCs from healthy donors using seven different flow cytometers. Additionally, human PBMCs were analyzed by fluorescence microscopy, imaging flow cytometry and high-content imaging (HCI). Flow cytometers differed significantly in their ability to detect ASC speck^+^ cells. Aria III, Astrios EQ, and Canto II performed best in separating ASC speck^+^ from diffuse ASC cells. Imaging flow cytometry and HCI provided additional insight into ASC speck formation based on image-based parameters. For optimal results, the ability to separate cells with diffuse ASC from ASC speck^+^ cells is decisive. Image-based parameters can also differentiate cells with diffuse ASC from ASC speck^+^ cells. For the first time, we analyzed ASC speck detection by HCI in PBMCs and demonstrated advantages of this technique, such as high-throughput, algorithm-driven image quantification and 3D-rendering. Thus, inflammasome activation by ASC speck formation can be detected by various technical methods. However, the results may vary depending on the device used.

**Supplementary Information:**

The online version contains supplementary material available at 10.1007/s10753-024-02141-z.

## Introduction

The term autoinflammation was introduced in 1999 by Daniel Kastner when investigating germline mutations in the TNF receptor [1, 2]. Autoinflammation is characterized by the formation of an inflammasome. Inflammasomes can be formed by myeloid cells in response to tissue injury, infection, and metabolic stress as part of the innate immune response [3, 4]. These signals are perceived by sensory proteins, which include members of the nucleotide-binding oligomerization domain (NOD), leucine-rich repeat (LRR)-containing proteins (NLR) family NLRP1, NLRP3, NLRC4 and the ab-sent-in-melanoma 2 (AIM2) protein as well as pyrin [5, 6]. With the exception of NLRP1 and NLRC4, these bind the adapter protein apoptosis-associated speck-like protein containing a caspase-reruitment domain (ASC) via its amino-terminal pyrin (PYD) domain. Upon PYD-PYD interaction the ASC, which is distributed throughout the entire cell in a resting state, is multimerizes and forms large helical fibrils, so-called ASC specks [7–9]. ASC speck formation is the hallmark of inflammasome activation and recruits caspases via their CARD domain. These caspases are autocatalytically activated, enabling them to cleave pro-interleukin (IL)-1β, pro-IL-18 and Gasdermin-D into their active forms [10]. Gasdermin-D then binds to the cell membrane, forms a 10–14 nm pore with 16 symmetrical protomers and causes cell death [11]. This inflammatory form of cell death is called pyroptosis [12].

Uncontrolled inflammasome activation can lead to autoinflammatory diseases, such as cryopyrin-associated periodic syndrome (CAPS) or familial Mediterranean fever (FMF) [1, 13, 14]. The role of inflammasome activation is becoming increasingly relevant in a variety of diseases such as COVID-19 [15], diabetes [16], gout [17, 18], human immunodeficiency virus (HIV)-1 infection [19, 20], juvenile idiopathic arthritis [21], sepsis [22] and multiple sclerosis [23].

The flow cytometry-based method, to identify ex vivo ASC speck formation was introduced by Sester et al. [24] and has recently been optimized by us [25] for clinical applications. A key advantage of flow cytometry is the ability to perform rapid qualitative and quantitative analysis even with small sample volumes, which is an important point when dealing with patient samples, most importantly from children. The limited number of cells makes traditional immunoblotting a challenge. Protein biochemistry is also more time consuming and less sensitive. Furthermore, flow cytometry is a reliable method for cell type-specific analysis and offers advantages over conventional manual fluorescence microscopy, which does not reliably quantify activated cells [26].

Novel methods such as imaging flow cytometry or high-content imaging (HCI) have these advantages and provide additional structural information by generating an image of each cell. However, different instruments use different components and software to calculate the detected events. Therefore, we investigated similarities and differences between several devices in their ability to detect ASC specks in human monocytes. Therefore, in the current study we compared different technical methods including different flow cytometers in their ability to detect ASC specks in human monocytes.

## Results

### Detections of ASC Speck-Positive Cells by Fluorescence Microscopy and Different Flow Cytometers

**Fig. 1:**
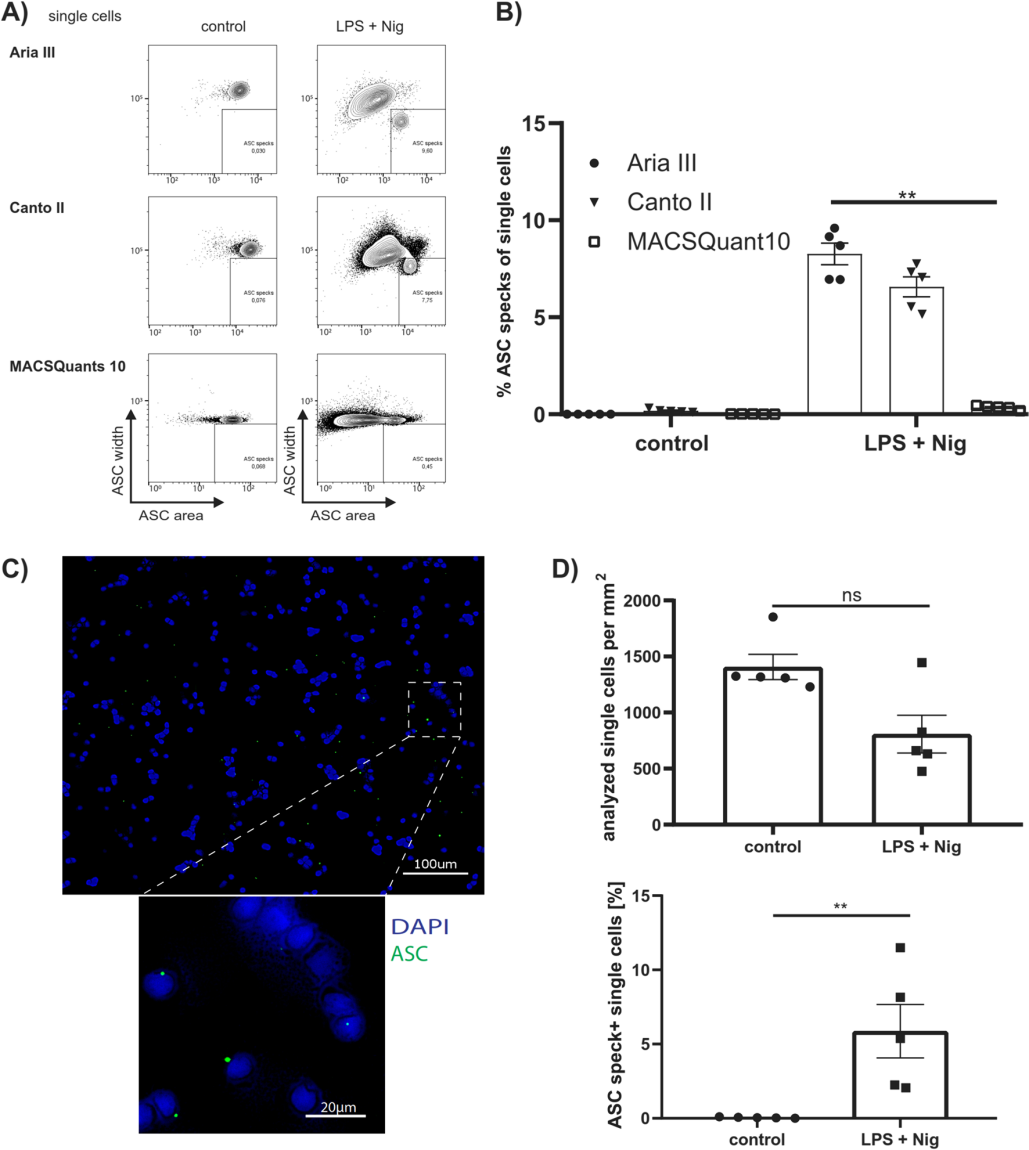
Comparison of flow cytometric and fluorescence microscopic ASC speck detection. (**A**) Representative contour plot of single cells from the same healthy donor measured by different flow cytometers. (**B**) Quantification of data from five samples of healthy donors, measured on different flow cytometers in two independent experiments. (**C**) Representative fluorescence image of stimulated PBMCs ASC (green) and DAPI (nuclei, blue) (scale bar 100 µm). (**D**) Quantification of analyzed single cells per square millimeter and ASC specks of either stimulated with LPS and Nig- or control PBMCs from five healthy donors. For (**B**) a Friedman test, followed by Dunn's multiple comparisons test and for (**D**) a Mann–Whitney-U test was performed. P-values *p < 0.05, **p < 0.01 and ***p < 0.001 were considered statistically significant. Data are shown as mean ± SEM.

Under resting conditions, ASC is distributed uniformly throughout the cell and rearranges under inflammasome activation into a single spot, the ASC speck. The pulse width value of the ASC fluorescence signal is an important parameter for flow cytometric detection of ASC speck^+^ cells [24]. Aggregated ASC specks have a smaller width signal and can thereby be distinguished from the uniformly distributed ASC. Each flow cytometer has a different ability to detect this change. The same samples from five healthy donors were measured on an Aria III, Canto II and MACSQuant10 (Fig. 1A-B).

Aria III and Canto II have distinguished the two ASC phenotypes, while the MACSQuant10 was not able to detect ASC speck^+^ cells. To further evaluate nascent results with an independent method, a traditional approach using immunofluorescence microscopy was applied as well. For cell and ASC speck detection high resolution merged images from each sample were acquired. Some of the stimulated cells have probably been lost due to the processing and additional washing steps. But even though in control samples more single cells per square millimeter were measured no ASC speck^+^ cells were detected. Compared to the flow cytometric detection of ASC specks, a similar relative number of ASC speck^+^ single cells can be detected in stimulated (100 ng/ml LPS for 4 h and 10 µM nigericin (Nig) for 20 min) samples (Fig. 1C-D). To ensure comparability with fluorescence microscopy, with has less channels than the flow cytometers, we neglected the flow cytometers ability to generate lineage-specific results and only considered ASC staining values related to single cells.

After we observed differences between various flow cytometers, we wanted to investigate these differences in greater detail. Therefore, PBMCs from healthy donors were analyzed in two independent experiments as control and stimulated with LPS and Nig. The same samples were measured on seven different flow cytometers (Aria III, MoFlo Astrios^EQ^, Attune NxT, Canto II, CytoFLEX LX, LSR II and MACSQuant 10) gated for CD14^+^ CD16^−^ monocytes and analyzed for their ASC signal (Fig. S1).

**Fig. 2:**
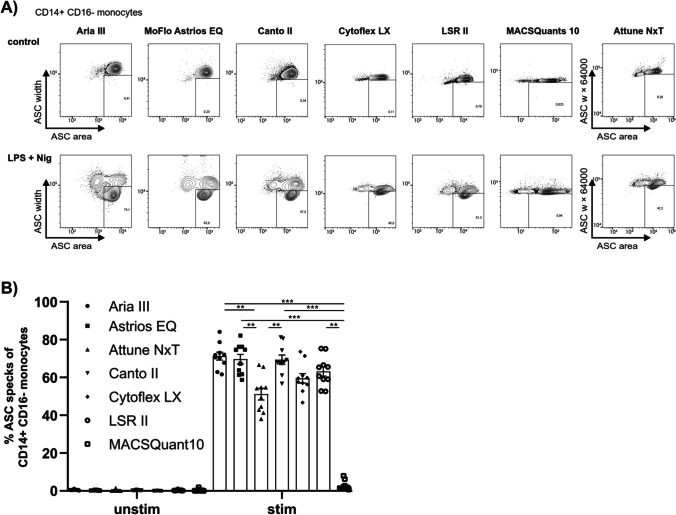
Separation of the ASC width signal depending on the flow cytometer device used. (**A**) Representative contour plot of CD14^+^CD16^−^ monocytes from the same healthy donor measured by different flow cytometers. (**B**) Quantification of data from ten samples of healthy donors, measured on different flow cytometers in two independent experiments. Friedman test was performed, followed by Dunn's multiple comparisons test. P-values *p < 0.05, **p < 0.01 and ***p < 0.001 were considered statistically significant. Data are shown as mean ± SEM.

Although the cytometers were equipped with different laser, filter and detector combinations, some of the contour plots of the same samples appeared more similar to each other on different cytometers than others (Fig. 2A). After signal detection, the electric signal was converted to a digital number. The width signal was calculated by the area divided by height and multiplied by 64,000 in the FACSDiva software (BD Biosciences) or multiplied by 1024 in CytExpert software to digitally display the plot. The Attune NxT processes the data by the field programmable gate array (FPGA), which simultaneously calculates pulse height, area, and width once the pulse exceeds the threshold value. A derived parameter was calculated using FlowJo software (BD Biosciences) to enable a standardized appearance and a comparative analysis (Fig. S2).

As expected, ASC speck^+^ monocytes were not detected in control samples. Within the same stimulated samples, the number of ASC speck^+^ monocytes significantly differed between the flow cytometers (Fig. 2B). The two cell sorters (Aria III and MoFlo Astrios^EQ^) measured the highest numbers, followed by Canto II which all detected significantly more ASC speck^+^ monocytes compared to MACSQuant 10. Furthermore, these flow cytometers detected significantly more ASC speck^+^ monocytes than Attune NxT. The reason was an inferior ability to detect the decrease of the ASC width signal. For samples from MACSQuant 10, this was particularly evident. There was, a horizontal rather than a vertical shift of the ASC signal in LPS and Nig-stimulated cells. Thus, ASC specks could not be identified in CD14^+^ CD16^−^ monocytes using MACSQuant 10. To determine the instrument-specific detection limits for small particles, we analyzed green fluorescent beads with diameters ranging from 0.5 μm to 2.0 μm. The results revealed a broader distribution for the 0.5 μm bead population compared to the 2.0 μm bead population across all devices tested (Fig. S3). Notably, the MACSQuant10 displayed the widest distribution for all bead populations.

**Fig. 3:**
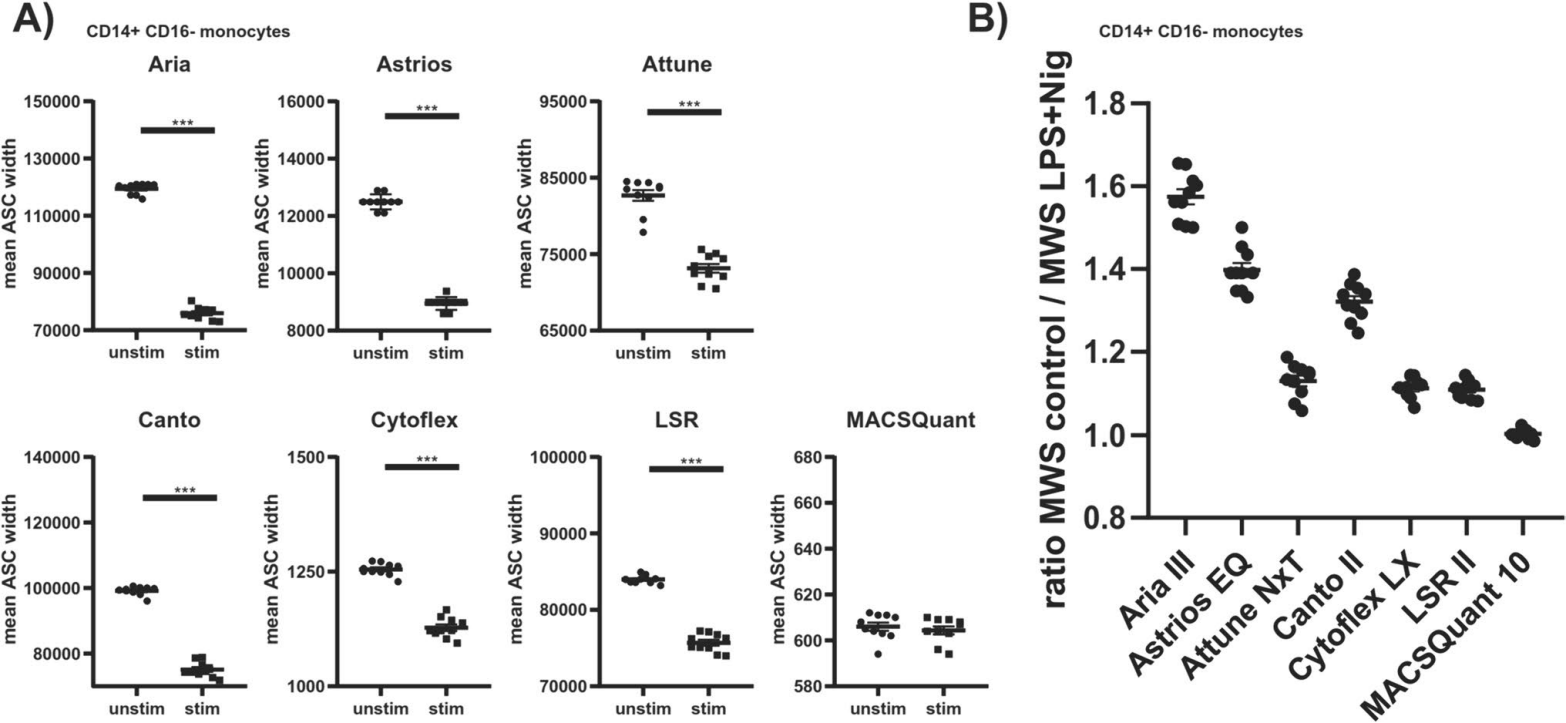
Ratio of mean ASC width signal from control and stimulated cells indicated the separation of ASC speck^+^ cells from the uniformly distributed ASC population. (**A**) Quantification of mean ASC width of CD14^+^CD16^−^ monocytes from ten samples of healthy donors were either unstimulated (control) or stimulated with 100 ng LPS for 4 h and 10 ng nigericin for 20 min, as measured in two independent experiments. **(B)** The ratio between the mean ASC width signals (MWS) from control against LPS and Nig stimulated PBMCs. A Mann–Whitney-U test was performed. P-values *p < 0.05, **p < 0.01 and ***p < 0.001 were considered statistically significant. Data are shown as mean ± SEM.

To better compare the ability of instruments to detect the change in ASC width signal we compared the mean ASC width signal of CD14^+^ CD16^−^ monocytes from control and stimulated samples. On all devices the mean ASC width signal (MWS) was significantly reduced in LPS and Nig stimulated monocytes compared to the control group, except for MACSQuant 10 (Fig. 3A). This is consistent with data comparing percentages of ASC speck^+^ monocytes from all CD14^+^ CD16^−^ monocytes. To evaluate them according to their ability to separate the ASC width signal, a ratio, between the mean ASC width of control and stimulated samples, was formed (Fig. 3B). A larger ratio indicates a better separation between these two ASC phenotypes. For CD14^+^ CD16^−^ monocytes Aria III, MoFlo Astrios^EQ^ and Canto II had the highest ratio, followed by Attune NxT, CytoFLEX LX and LSR II. The MACSQuant 10 ratio was 1.003, as there was no difference in mean ASC width between control and stimulated samples.

This indicates that the detection of the change in ASC width signal is critical for the detection of ASC speck^+^ monocytes. In the same samples Aria III, AstriosEQ and Canto II detected the highest amount of ASC speck^+^ monocytes and the best separation between the ASC specks and the diffuse ASC population.

### ASC Speck-Positive Cell Quantification by Image Cytometry-Based Parameters

**Fig. 4:**
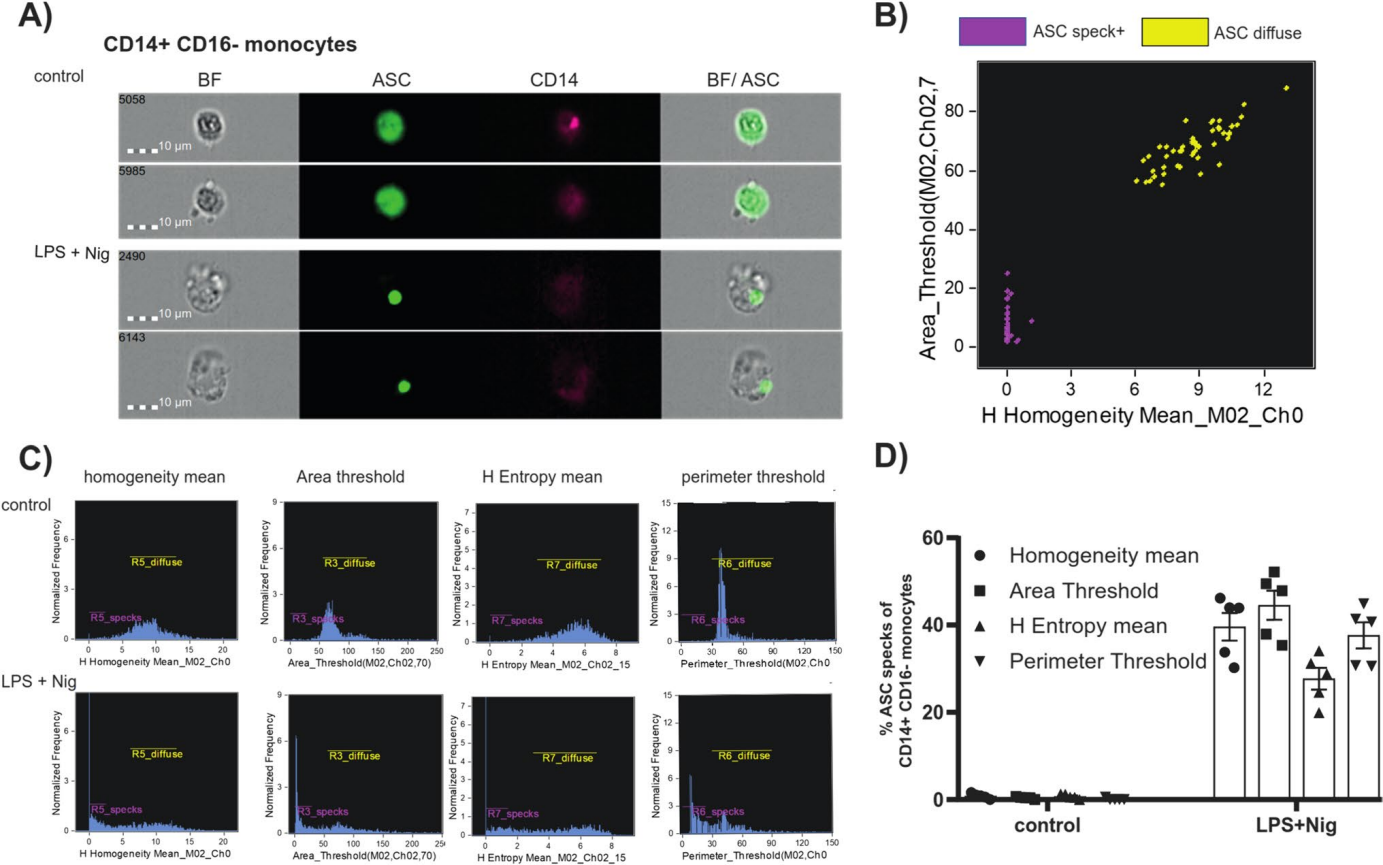
ASC speck detection by image-based parameters. (**A**) Representative images of focused CD14^+^CD16^−^ monocytes were selected, showing: BF (brightfield), ASC (green), CD14 (pink) and a composite image of the BF with fluorescence dye (ASC). (**B**) Representative dot-plot of the top two features suggested by Feature Finder software: H homogeneity mean versus area threshold showing the separation of the assigned “truth populations”. (**C**) Histograms of CD14^+^ CD16^−^ monocytes either stimulated with LPS and Nig or left untreated according to the top four features: H homogeneity mean; area threshold; H entropy mean; perimeter threshold. (**D**) Quantification of data from five healthy donors by these image-based parameters. Data are shown as mean ± SEM.

We next inferred whether image cytometry-based parameters differed from flow cytometric detected values. Therefore, five samples used for flow cytometer comparison were also acquired by an imaging flow cytometer (Amnis ImageStreamX Mk I). In addition to the fluorescence signal, this instrument captures a microscopic image of each cell (Fig. 4A). For analysis, single and focused CD14^+^ CD16^−^ monocytes were used.

First, “truth populations” have been defined following the feature finder wizard instructions. At least 50 CD14^+^ CD16^−^ monocytes representing phenotypical differences (diffuse ASC and ASC specks) were manually selected. The IDEAS software determined the best features, to separate the two phenotypes. The most suitable features were H homogeneity mean and area threshold (Fig. 4B, truth populations). An increase in ASC speck^+^ monocytes could be detected for each selected feature (H homogeneity mean; area threshold; H entropy mean; perimeter threshold) in LPS and Nig stimulated samples compared to controls, with slightly different numbers of ASC speck^+^ cells (Fig. 4C-D). The identified top features showed slightly fewer ASC speck^+^ cells compared to the flow cytometry-based analysis, with the entropy mean detecting the lowest amount of ASC speck^+^ cells. Hence, it is possible to use image-based parameters to detect of ASC speck^+^ monocytes with the advantage of additional information about the size of ASC specks or structural changes within the cells.

### Analyzing ASC Speck Formation by High-Content Imaging

**Fig. 5:**
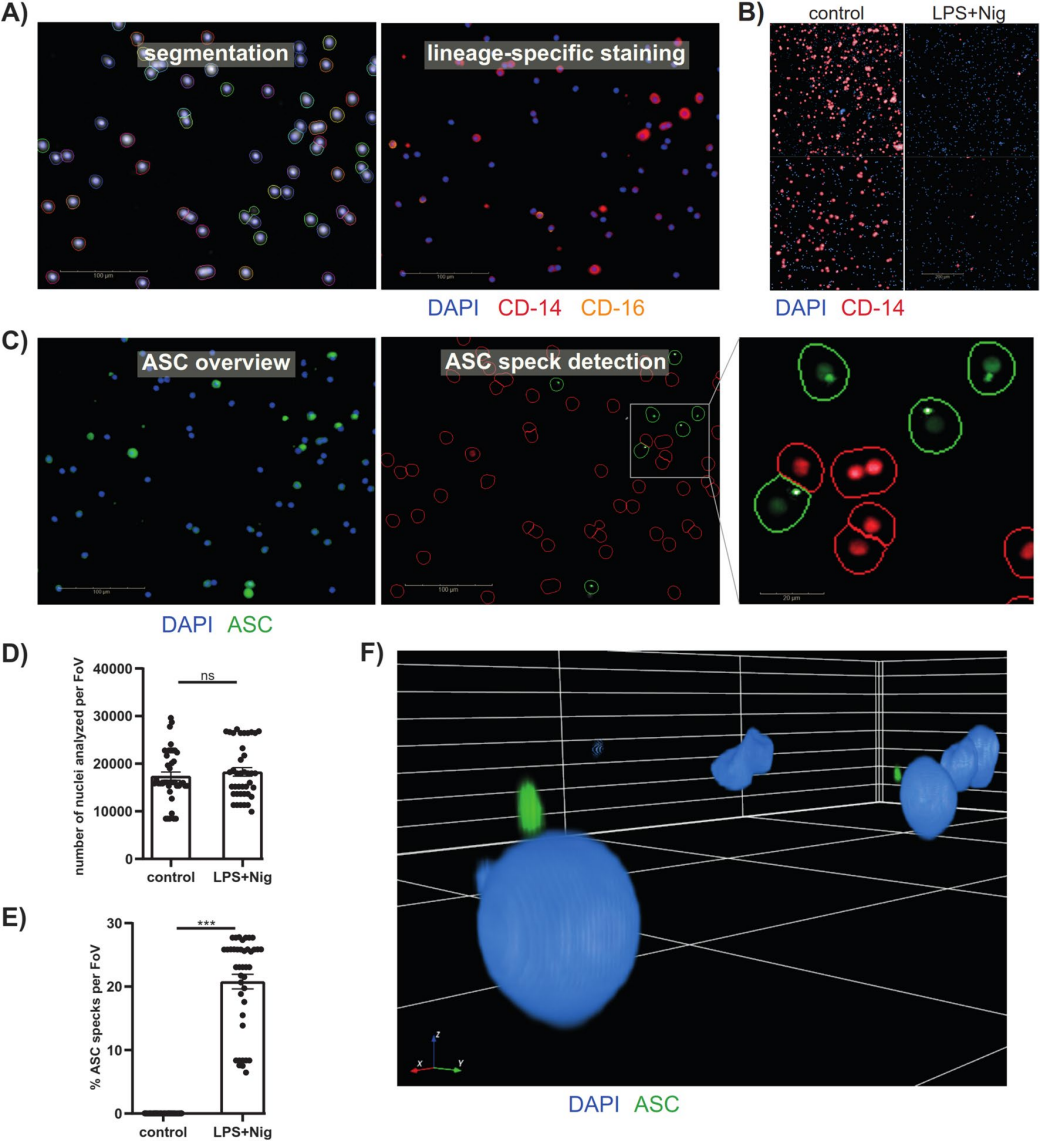
Algorithm-driven image quantification and 3D-rendering of ASC specks by high-content imaging. **(A**) Representative fluorescence image for cell segmentation based on DAPI signal considering the cytoplasm and lineage-specific staining of stimulated PBMCs; (CD14 (red), CD16 (orange) and DAPI (nuclei, blue) (scale bar 100 µm). **(B)** CD14 signal from two FoVs of control and LPS + Nig stimulated PBMCs. **(C)** Algorithm-driven ASC speck detection in stimulated PBMCs; ASC (green) DAPI (nuclei, blue) (scale bar 100 µm). **(D)** Quantification of analyzed nuclei and (E) ASC specks from four FoVs of either stimulated with LPS and Nig or control PBMCs from ten healthy donors, as measured in two independent experiments. (F) 3D image from 70 z-stacks of stimulated PBMCs measured by a 63 × water immersion objective ASC (green) and DAPI (nuclei, blue). A Mann–Whitney-U test was performed. P-values *p < 0.05, **p < 0.01 and ***p < 0.001 were considered statistically significant. Data are shown as mean ± SEM.

HCI enables single-cell measurements in great quantity while accurately detecting different phenotypes. This method involves a high degree of computerized automation, allowing users to accelerate repetitive operations and remove human bias from each step. As a result, imaged data can be acquired using the exact same settings and processed and analyzed in batch mode using algorithm-driven image quantification and object segmentation within minutes. Data obtained include information such as brightness, fluorescence signal, quantity, size, shape, and relative or absolute location for regions of interest. The rationale for comparison was to use a non-flow-cytometry method to quantitatively assess ASC speck formation. This was proposed because flow cytometry-based detection relied on time-of-flight / signal width analysis, while fluorescence microscopy analysis is more straightforward to unambiguously identify specks. A portion of the same anti-ASC AL177-incubated PBMCs, which were used for the flow cytometer comparison and imaging flow cytometer detection, was utilized and stained with different lineage-specific antibodies (CD14-APC and CD16-PE/Dazzle-594) and anti-rabbit-IgG-AF488 for ASC detection. Computer-driven cell segmentation was performed based on the DAPI signal (nuclei), considering the cytoplasm that was reflected in a digital phase contrast channel. (Fig. 5A). Digital phase contrast allows segmentation of image areas adjacent to cellular nuclei based on motorized-controlled de-focusing of the image in the brightfield channel and calculation of a virtual image of accumulated contrasts. For quantification, CD14^+^ CD16^−^ monocytes were detected and quantified for each field of view (FoV). This was done by calculating the mean intensities of two antibody staining’s in the cytoplasm segmented for all segmented nuclei identified in the previous step. Then, thresholds were set using the selected population building block to gate monocyte populations based on their CD14 and CD16 staining intensities. The CD14 signal was decreased in LPS and Nig stimulated PBMCs compared to controls (Fig. 5B). Similar to flow cytometry, ASC specks were detected by their increased fluorescence signal relative to the surrounding area. For quantification, algorithm-driven detection of the maximum pixel intensity in the cell region was performed (Fig. 5C) using the “find micronuclei” building block implemented in the software. Cells with a high pixel intensity for the ASC signal were selected (labeled in green), while ASC speck-negative cells were excluded (labeled in red) using the selected population building block preloaded with binary information on ASC speck presence within each object (cell).

For quantification, four FoV from each sample were analyzed for the number of nuclei (Fig. 5D) and ASC speck^+^ cells (Fig. 5E). Due to the greatly reduced CD14 signal in stimulated PBMCs, hampering the unambiguous identification and enumeration of CD14^+^ monocytes, the calculation of the relative number of ASC speck^+^ cells within CD14^+^ CD16^−^ monocytes was not feasible. Therefore, percentage of ASC speck^+^ cells was related to all cells per FoV. The FoV showed no difference in the number of analyzed nuclei but detected a significant increase of ASC speck^+^ cells in LPS and Nig stimulated samples compared to controls. Unlike imaging flow cytometry, HCI can also acquire Z-stacks. Using 3D-rendering software, detailed cell images and localization of ASC specks were visualized **(**Fig. 5F). Hence, even complex structures or colocalization can be easily revealed.

The HCI is superior to other techniques in that it can capture thousands of samples per day with high quality microscopy. ASC specks can be detected by maximum pixel intensity, and image-based parameters can be used to indicate the relative position of ASC specks within the cell or to analyze changes in cell morphology.

## Discussion

Detection of ASC speck^+^ cells directly in the whole blood or after PBMC isolation is a reliable method to analyze human samples [24, 25, 27]. Autoinflammatory diseases, such as FMF or CAPS are very rare, therefore, samples are often collected in multicenter studies. Recent publications showed that blood samples should be processed within two hours after venipuncture to avoid unspecific ASC speck formation [25]. This complicates sample transport and warrants sample analysis close to the respective study site, making harmonization of devices such as flow cytometers for standardized sample acquisition difficult in multicenter studies.

In 2015 Sester et al. compared three different flow cytometers (Canto II, LSR II, and Accuri C6 from BD Biosciences) to fluorescence microscopy [24]. Our study provides an update to this initial report by extending the comparison to seven flow cytometers, one imaging flow cytometer, and HCI device.

The pulse width value of the ASC fluorescence signal plays a crucial role in the detection of ASC speck^+^ cells, which is not typical in conventional flow cytometry, in which only the intensity of a fluorescence signal is measured. While the concepts of pulse-area, -width, and -height are simple, the implementation of pulse statistics differs between flow cytometers and is often proprietary information [28]. Various cytometers use different analog-to-digital converters to convert voltage value to digital value according to the bit depth [29]. Bit depth influence the resolution, which dictates the ability of flow cytometers to distinguish two different signal levels. The BD FACSDiva Software and MACSQuant10 perform sample acquisition with 18 bits (262,144 channels), Astrios EQ performs sample acquisition with 32-bits (4,294,967,296 channels), while Attune NxT perform sample acquisition with 16-bits (65,536 channels) [28, 29]. Although the Attune NxT has a lower bit rate than the MACSQuant10 it was able to detect the ASC specks. Having the highest bit depth, Astrios EQ could detect a similar amount of ASC speck^+^ cells compared to Aria III, which operates with 18-bits. Other cytometers operating with the BD FACSDiva Software detected a smaller amount of ASC speck^+^ cell, concluding that the bit depth of a flow cytometer does not infer its ability to detect ASC speck.

Most devices that we tested used a Gaussian beam, while the Astrios EQ and Attune NxT had a flat-top beam installed. The advantages of flat-top beams over Gaussian beams are a reduced heat-affected zone and more efficiently utilized laser energy. The steeper edge transitions in flat-top beams reduce measurement uncertainty and statistical variance. In addition to hydrodynamic cell focusing used by most cytometers, Attune NxT uses acoustic cell focusing to precisely align cells for faster detection rates. Although these are major hardware-related differences, Astrios EQ detected an amount of ASC speck^+^ monocytes similar to Aria III, while Attune NxT detected an amount of ASC speck^+^ monocytes similar to LSR II or Cytoflex LX. Therefore, the installed beam system does not indicate how well a device can detect the ASC specks.

The most probable explanation for the differences in performance is the variation in laser spot sizes and signal calculation formulas. It can be assumed that larger laser spot sizes exert a more pronounced influence on the width signal than smaller laser spot sizes. This hypothesis was supported by bead size measurements, which showed that smaller beads displayed a more dispersed population compared to larger beads. Beads measured by the MACSQuant10 demonstrated the greatest dispersion among all bead populations. Thus, bead size measurement could be a useful indicator for pre-selecting an appropriate device.

The percentage of detected ASC speck^+^ cells is critical for subsequent comparison of clinical specimens. Thus, no flow cytometer detected ASC speck^+^ cells in unstimulated samples, while differences in identifying ASC speck^+^ monocytes were significant between devices. However, the detected percentage might have also depended on gating strategies and subjective perception of the evaluator, hence, we introduced the mean ASC width signal as a better parameter for instrument comparison. Thus, using mean ASC width removes human bias in the gate setting for ASC speck^+^ cells. Both parameters show a similar rank order of tested flow cytometers, indicating that devices with a better separation of ASC width signal can detect more ASC speck^+^ monocytes.

Image-based analysis methods should be considered if additional information is required, such as that about the size of ASC specks or changes in the cellular structure. The detection of ASC specks by imaging flow cytometry was the subject of several publications [30–32]. We identified the same top four features as Lage et al. [31], although in a different order, to distinguish ASC speck^+^ monocytes from those with diffuse ASC. Nagar et al. compared the number of ASC speck^+^ primary monocytes measured by imaging flow cytometry and flow cytometry (LSR II) [30]. A 1.5—twofold higher frequency of ASC speck^+^ cells was measured by flow cytometry. The data of our study also showed a lower amount of ASC speck^+^ monocytes using imaging flow cytometry compared to most flow cytometers tested in our study. This result might have occurred because imaging flow cytometry allows the removal of false-positive events based on image information, a lacking option in traditional flow cytometry.

Although conventional flow cytometry can assay large numbers of cells, it is limited by its inability to detect colocalized enzymatic activity and evaluate the speck morphology. Thereby, image-based parameters can be used to distinguish between diffuse ASC and ASC specks. A detailed description of these features and their differentiation for ASC phenotypes has been described by Lage et al. [31]. Image-based parameters also visualize the impact of other components of the inflammasome on the ASC specks. The presence of NLRP3 reduces the size of ASC specks and they are negatively correlated to the presence of active caspase-1 [30]. However, both imaging flow cytometry and conventional flow cytometry have limitations, especially when analyzing tissue samples from inflamed tissue. The process of disrupting the tissue for subsequent analysis can potentially damage fragile ASC speck^+^ cells that are undergoing pyroptosis. Acquisition of z-stacks to visualize colocalization or analysis of tissue samples is typically performed by confocal microscopy, which is very time-consuming.

To address the above-mentioned problem, we introduced the HCI, which is an imaging method with high quantity and quality. For the first time, we performed ASC speck detection by HCI in human PBMCs. In addition to primary cells, complex 3D models and tissues can be examined using HCI [33]. Many samples can be conveniently measured in 96- or 384-well plates at the single-cell level and undergo algorithm-driven analysis for phenotypic structures in the batch mode. This makes this method particularly suitable for detailed analysis of many samples with limited cell numbers, such as pediatric blood samples. In contrast to flow cytometry, the HCI does not use differences in area and width signal but identifies ASC specks based on their increased luminosity compared to the diffuse ASC. Maximal pixel intensity proved to be a reliable parameter to distinguish the two phenotypes. However, LPS and Nig stimulation led to decreased CD14 expression, which was also observed by flow cytometry. This reduction prevented a proper assignment of CD14^+^ CD16^−^ cells for HCI, at least with the antibodies and fluorochromes utilized in this study. This limitation could be overcome in the future by generating comprehensive analysis masks and using machine learning along with improved staining protocols. Therefore, direct comparison with flow cytometry data is limited.

A unique feature of the HCI is the ability to generate high quantity and quality of z-stacks, which might not only be suitable to detect the location of ASC specks within cells or tissues but could also visualize colocalization with a sensor protein and caspase-1. However, such analysis remains the subject of future studies.

## Materials and Methods

### Reagents

The following reagents and chemicals were used: ultrapure lipopolysaccharide (LPS) (cat. number: tlrl-3pelps, from E. coli 0111:B4, Invitrogen, Waltham, MA, USA), nigericin (Nig), trypan blue (all Sigma-Aldrich, St. Louis, MO, USA), RPMI1640 and Flow Cytometry Sub-micron Particle Size Reference Kit (Thermo Fisher Scientific, Waltham, MA, USA), Cytofix/Cytoperm Fixation/Permeabilization Kit (BD Biosciences, Franklin Lakes, NJ, USA), Ficoll, and PBS (all PAN-Biotech, Aidenbach, Germany). The following antibodies were used: rabbit-polyclonal anti-ASC (AL177, Adipogen, San Diego, CA, USA), anti-rabbit-IgG-AlexaFluor488 (Invitrogen, Waltham, MA, USA), and lineage-specific antibodies CD3-PerCP, CD4-APC, CD14-APC-Cy7, CD14-APC, CD16-PE-Cy7, CD16-PE/Dazzle-594 and DAPI (4',6-Diamidino-2-Phenylindole, Dilactate) (BioLegend, San Diego, CA, USA).

### Patient Acquisition and Blood Sample Processing

All healthy donors were recruited at the University Medicine Greifswald (Germany) and included in the study after providing written informed consent. This study was conducted according to the Declaration of Helsinki, and the protocol was approved by the local ethics committee (BB032/21). Samples from eight different healthy donors were analyzed in two independent experiments. Lithium-heparin blood collection tubes were used to obtain blood samples. Peripheral blood mononuclear cells (PBMCs) were isolated from blood using density gradient centrifugation 460 g for 20 min with low acceleration and deceleration. Afterwards they were collected in PBS medium and washed twice with PBS at room temperature (400 g for 10 min). The respective pellet was re-suspended, and the cell count was calculated using trypan blue and a Neubauer counting chamber, as previously described [25]. PBMCs were primed with 100 ng/ml LPS for 4 h in RPMI 1640 (PAN Biotech) and nigericin (Nig) was added at a final concentration of 10 µM for 20 min at 37°C.

### Flow Cytometry

According to the manufacturer´s instructions, unstimulated and stimulated PBMCs were fixed on ice with 250 µl Cytofix (BD Biosciences) for 20 min. After washing with the permeabilization medium Cytoperm™ (BD Bioscience), fixed samples were kept overnight at 4 °C in the permeabilization medium. For longer storage of fixed cells, we recommend to wash and then store samples in PBS. The cells were stained the next day with anti-ASC (AL-177, 1:1000) and lineage-specific antibodies (1:200) as previously described [21, 25]. The same samples were measured on the same day on different devices (Aria III, Canto II, LSR II (BD Biosciences), MACSQuant10 (Miltenyi Biotec, Bergisch Gladbach, Germany); MoFlo Astrios^EQ^; CytoFLEX LX (Beckman-Coulter, Brea, CA, USA), Attune NxT (Thermo Fisher Scientific). The flow cytometer configurations can be found in Table S1. To enable a standardized appearance along all flow cytometers, a derived parameter was calculated for the Attune NxT using FlowJo software (BD Biosciences). In consultation with the company support the derived parameter was calculated ASC-w × 64,000. Samples on all devices were examined using the same gating strategy (Fig. S1). First, debris was excluded, then single cells were determined and gated on CD3^−^ cells. Finally, the CD14^+^ CD16^−^ cells were analyzed for their ASC signal.

### Immunocytochemistry and Fluorescence Microscopy

Shandon cytospin 4 (Thermo Scientific) was used to deposit freshly isolated or stimulated PBMCs into a clearly defined area of a “SuperFrost® plus” glass slide (R. Langenbrinck, Emmendingen, Germany). Fixation was done with ice cold methanol at − 20°C for 15 min. The glass slides were then stored at − 80°C until further usage. For staining, glass slides were warmed up fast and washed three times with buffer (PBS containing 0.2% BSA, 0.05% saponin, and 0.1% NaN3). Cells were edged with a liquid-repellent slide marker (Plano GmbH), before incubating with Image-iT™ FX Signal Enhancer (Invitrogen) at room temperature (RT) for 30 min, to prevent flooding. After washing for 5 min on a tilt shaker with buffer, incubation with rabbit anti-ASC (1:1000, AL177 from AdipoGen) took place at 4 ^◦^C in a humid environment overnight. Slides were washed three times on a tilt shaker with buffer and incubated with anti-rabbit-IgG-AlexaFluor488 (1:200) in the dark at RT for 1 h. The glass slides were washed again three times and incubated with 3 µM DAPI for 5 min. After a final washing step, coverslips were mounted on slides with the Dako fluorescence mounting medium (Agilent, Santa Clara, California, USA). Images were captured on a BZ-9000 fluorescence microscope (Keyence, Osaka, Japan) at constant exposure times (DAPI 1/30s; AlexaFluor488 1/5s) and magnification (60x). Array imaging was used to create 225, z-stacked field of view single images. Overlapping image patches were then merged. The full sample image was partitioned into four separate cell areas for subsequent analysis by hand. Cell and ASC speck detection were performed utilizing the built-in tools of the QuPath software [34]. Cells were classified based on mean DAPI intensity, shape and size. ASC specks were detected by AlexaFluor488 intensity thresholding and subsequent object creation, with size-based exclusion. Detected cells and specks were divided by respective annotated region size to avoid underestimation by smaller spin area and staining artifacts.

### Imaging Flow Cytometry

PBMCs from five donors were acquired using an Amnis ImageStreamX MK I (Cytek Biosciences, Fremont, CA, USA). The imaging flow cytometer equipped with 405-, 488-, 561- and 658-nm lasers acquired samples at 40 × magnification, 66 mm/s, and high resolution. Single-color compensation controls were acquired using the INSPIRE software. Channel 2 (ASC), channel 5 (CD3), channel 11 (CD4), channel 12 (CD14) and channel 6 (CD16). A 785-nm laser was used for side scatter (SSC) detection and a LED for brightfield. All samples were acquired as raw image files (.rif). Compensation controls and all samples were analyzed by Image Data Exploration & Analysis Software (IDEAS). The compensation matrix was applied to PBMCs. The cells were analyzed by features, image-based parameters such as size, texture, shape and masks, and image-based parameters for a region of interest, such as the entire cell or specific cell compartments, as described in Lage et al. [31].

### High-Content Imaging Analysis

Anti-ASC AL177-incubated (1:1000 from AdipoGen, San Diego, CA, USA) PBMCs were washed with 1 mL of the permeabilization medium at 600 g for 8 min. The supernatant was removed, and the cells were incubated with a permeabilization medium containing anti-rabbit-IgG-AlexaFluor488 (1:750 from Invitrogen, Waltham, MA, USA) (AF488) and PBMCs lineage-specific fluorochrome-labeled monoclonal antibodies targeting CD14 (-APC) and CD16 (-PE/Dazzle-594) (1:20 from BioLegend, San Diego, CA, USA) on ice for 30 min. The cells were washed again with 1 mL of the permeabilization medium at 600 × g for 8 min, and DAPI (3 µM) was added. The cells were added to glass-bottom HCI plates (Eppendorf, Hamburg, Germany). Fluorescence microscopy was performed using an Operetta CLS HCI system (PerkinElmer, Hamburg, Germany). Excitation sources were four LEDs (365 nm, 130 mW; 475 nm, 110 mW; 550 nm, 170 mW; 630 nm, 250 mW), and emission filters were 465 ± 35 nm (DAPI), 525 ± 25 nm (ASC), 610 ± 40 nm (CD16), and 708 ± 52 nm (CD14). For quantitative analysis, images were acquired using a 20 × air objective (NA = 0.4; Zeiss, Jena, Germany), and at least 25 fields of view were taken per well. For the generation of 3D images, a 63 × water immersion objective (NA = 1.15; Zeiss, Germany) was used to acquire at least 70 z-stacks. Algorithm-driven image quantification and 3D rendering were performed using Harmony software 4.9 (PerkinElmer).

### Statistical Analysis

Data were presented as mean ± SEM from the number of experiments indicated. Multiple comparisons of nonparametric paired samples were performed by a Friedman test, followed by Dunn's multiple comparisons test. A Mann–Whitney-U test was performed as appropriate to detect a difference between the two groups. P-values *p < 0.05, **p < 0.01, or ***p < 0.001 were considered statistically significant. Statistical analysis was calculated using Prism software (GraphPad Software, San Diego, CA, USA).

## Conclusions

All devices are individual, and their features are adapted to the user’s needs. Which one is best suited depends on the research question since it might also be needed to evaluate other components of the inflammasome cascade to get the most complete picture. A flow cytometer is needed for the specific detection of ASC speck in PBMCs, which can detect the width change from diffuse ASC cells. In our screening, Aria III, AstriosEQ, and Canto II provided the best results. For a simple statement of whether ASC specks are present or not, a suitable flow cytometer is sufficient. If additional information about the size of ASC specks or structural changes within the cell is required, then image-based analysis methods should be applied. Acquiring these data can be achieved by imaging flow cytometry (high quantity) or confocal microscopy (high quality), while the HCI combines both features.

## Supplementary Information

Below is the link to the electronic supplementary material.

Supplementary file1 (PDF 331 KB)

## Data Availability

Data is provided within the manuscript or supplementary information files.
