# Supplementary material for: Comparative Analysis of Canonical Inflammasome Activation by Flow Cytometry, Imaging Flow Cytometry and High-Content Imaging
Source: Inflammation. 2024 Sep 10;48(3):1513–24. doi: 10.1007/s10753-024-02141-z (PMC12234628; doi:10.1007/s10753-024-02141-z)
Supplement: Supplementary file 1 — Supplementary file1 (PDF 331 KB) [file 10753_2024_2141_MOESM1_ESM.pdf]

SUPPLEMENT

Figure S1:

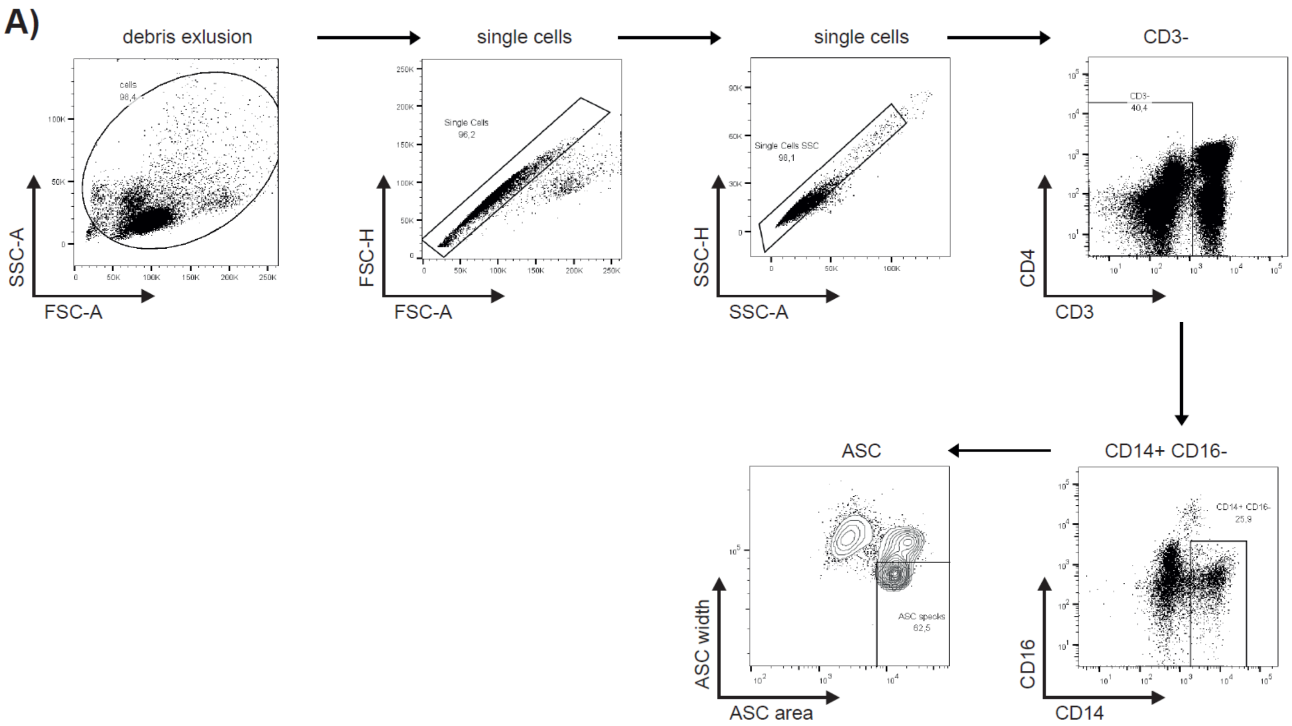

Figure S1: Representative gating strategy of LPS and nigericin stimulated PBMCs.

Figure S2:

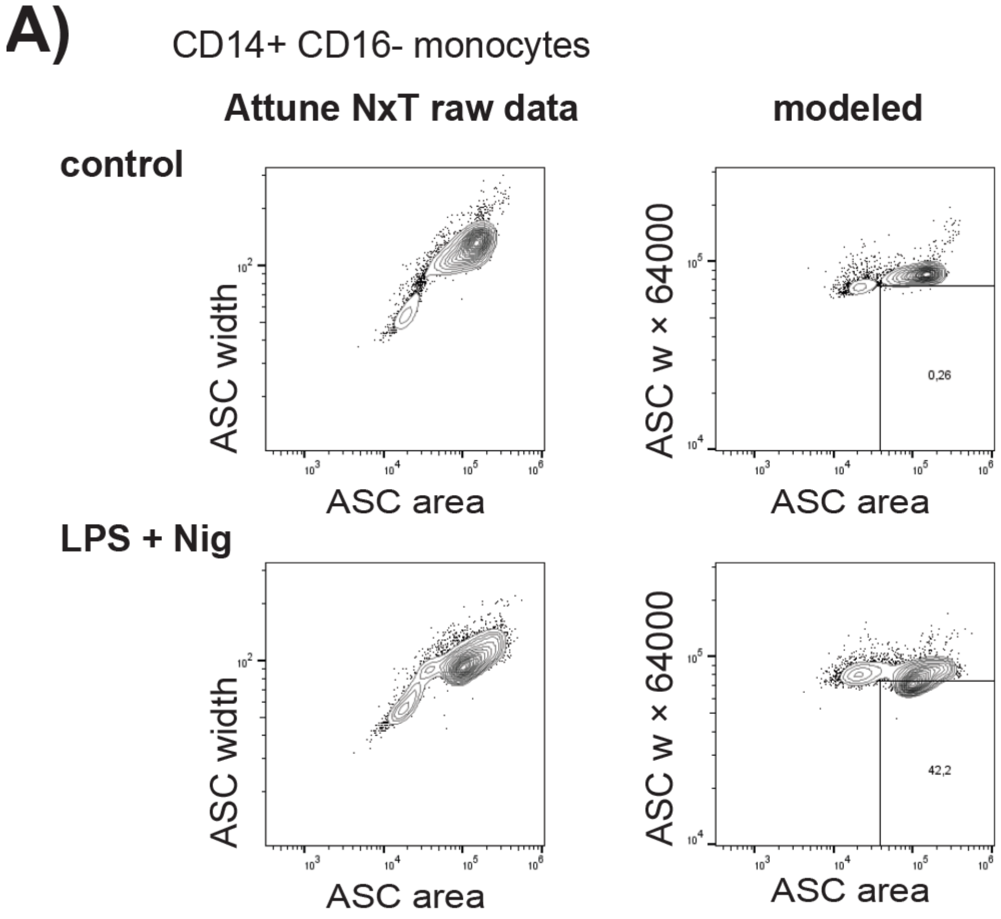

Figure S2: Representative data modeling of Attune NxT (.fcs)-files with FlowJo software to standardize the appearance and enable comparative analysis.

Figure S3:

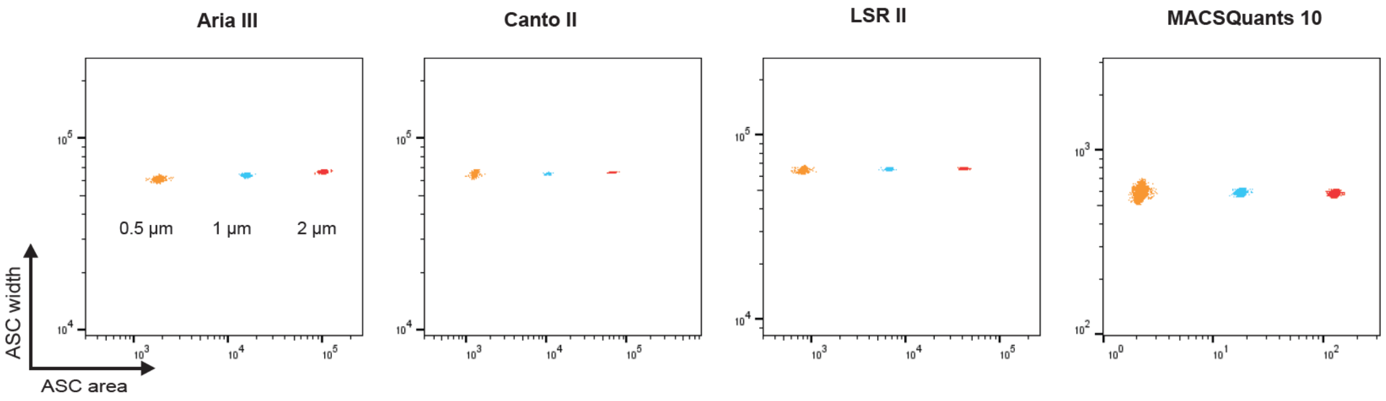

Figure S3: Representative dot plots of green fluorescent beads with diameters ranging from 0.5 µm to 2.0 µm were measured on different flow cytometers.

Table S1: Overview of the flow cytometer configurations.

Device	Aria III	Astrios EQ	Attune NxT	Canto II	Cytoflex LX	LSR II	MACSQuant10
Laser type	Gaussian beam	Flat-top beam	Flat-top beam	Gaussian beam	Flat-top beam	Gaussian beam	Gaussian beam
Built-in laser (power)	355 nm (15 mW)	355 nm (100 mW)	/	/	355 nm (20 mW)	/	/
	405 nm (50 mW)	405 nm (55 mW)	405 nm (50 mW)	405 nm (30 mW)	405 nm (80 mW)	405 nm (100 mW)	405 nm (40 mW)
	488 nm (20 mW)	488 nm (165 mW)	488 nm (50 mW)	488 nm (20 mW)	488 nm (50 mW)	488 nm (100 mW)	488 nm (30 mW)
	561 nm (50 mW)	561 nm (200 mW)	561 nm (50 mW)	/	561 nm (30 mW)	561 nm (50 mW)	/
	633 nm (18 mW)	633 nm (100 mW)	638 nm (100 mW)	633 nm (17 mW)	638 nm (50 mW)	640 nm (40 mW)	640 nm (20 mW)
Emission filter							
AF488	530 ± 30 nm	526 ± 52 nm	530 ± 30 nm	530 ± 30 nm	525 ± 40 nm	530 ± 30 nm	525 ± 50 nm
PerCP	695 ± 40 nm	710 ± 45 nm	695 ± 40 nm	>670 nm	690 ± 50 nm	710 ± 50 nm	655–730 nm
PE-Cy7	780 ± 60 nm	795 ± 70 nm	780 ± 60 nm	780 ± 60 nm	763 ± 43 nm	780 ± 60 nm	750 nm LP
APC	660 ± 20 nm	671 ± 30 nm	670 ± 14 nm	660 ± 20 nm	660 ± 10 nm	670 ± 14 nm	655–730 nm
APC-Py7	780 ± 60 nm	795 ± 70 nm	780 ± 60 nm	780 ± 60 nm	763 ± 43 nm	780 ± 60 nm	750 nm LP
analog-to-digital converter	18 bit	32 bit	16 bit	18 bit	24 bit	18 bit	18 bit
sampling frequency in MHz	10	100	20	10	25	10	n.a.*

* lasers are not pulsed, but have a constant wave
